# Diagnosis and Management of Kaposi Sarcoma-Associated Herpesvirus Inflammatory Cytokine Syndrome in Resource-Constrained Settings: A Case Report and an Adapted Case Definition

**DOI:** 10.3390/tropicalmed9120307

**Published:** 2024-12-16

**Authors:** Tapiwa Kumwenda, Daniel Z. Hodson, Kelvin Rambiki, Ethel Rambiki, Yuri Fedoriw, Christopher Tymchuk, Claudia Wallrauch, Tom Heller, Matthew S. Painschab

**Affiliations:** 1 Lighthouse Clinic Trust, Kamuzu Central Hospital Area, 33 Mzimba Street, P.O. Box 106, Lilongwe, Malawi; krambiki@lighthouse.org.mw (K.R.); erambiki@lighthouse.org.mw (E.R.); cwallrauch@lighthouse.org.mw (C.W.); theller@lighthouse.org.mw (T.H.); 2 Division of Internal Medicine—Pediatrics, David Geffen School of Medicine at UCLA, Los Angeles, CA 90095, USA; dhodson@mednet.ucla.edu; 3 Department of Pathology and Lab Medicine, University of North Carolina School of Medicine, Chapel Hill, NC 27599, USA; yuri.fedoriw@unchealth.unc.edu; 4 Institute for Global Health and Infectious Diseases, University of North Carolina School of Medicine, Chapel Hill, NC 27599, USA; 5 Lineberger Comprehensive Cancer Center, University of North Carolina at Chapel Hill, Chapel Hill, NC 27599, USA; matthew_painschab@med.unc.edu; 6 Division of Infectious Diseases, David Geffen School of Medicine at UCLA, Los Angeles, CA 90095, USA; ctymchuk@mednet.ucla.edu; 7 Institute of Infectious Diseases and Tropical Medicine, LMU University Hospital, LMU Munich, 80802 Munich, Germany; 8 International Training and Education Center for Health, University of Washington, Seattle, WA 98195, USA; 9 Adult Oncology Division, UNC Project Malawi, Tidziwe Centre, Private Bag A-104, Lilongwe, Malawi

**Keywords:** Kaposi sarcoma, Kaposi sarcoma-associated herpesvirus, Kaposi sarcoma-associated herpesvirus inflammatory cytokine syndrome, HIV, AIDS, resource-constrained setting

## Abstract

Kaposi sarcoma-associated herpes virus (KSHV), also known as human herpes virus 8 (HHV-8), is the primary etiologic cause of Kaposi sarcoma (KS) and KSHV Inflammatory Cytokine Syndrome (KICS). Patients with KICS demonstrate symptoms of systemic inflammation, high KSHV viral load, elevation of inflammatory markers, and increased mortality. Management requires rapid diagnosis, treatment of underlying HIV, direct treatment of KS, and addressing the hyperimmune response. While a case definition based on clinical presentation, imaging findings, laboratory values, KSHV viral load, and lymph-node biopsy has been proposed, some of the required investigations are frequently unavailable in resource-constrained settings. Due to these challenges, KICS likely remains underdiagnosed and undertreated in these settings. We report a case of a 19-year-old woman living with HIV, and intermittent adherence to her ART, who presented with hypotension and acute hypoxemic respiratory failure. She was found to have high KSHV and HIV viral loads, low CD4 count, anemia, thrombocytopenia, hypoalbuminemia, and elevated inflammatory markers. On bedside ultrasound, she was found to have bilateral pleural effusions, ascites, an enlarged spleen, and hyperechoic splenic lesions. The diagnosis of KICS was made based on this constellation of findings. Weighing the risk and benefits of steroid administration in KS patients, the patient was successfully treated by the continuation of ART and the initiation of paclitaxel chemotherapy and steroids. We propose an adapted case definition relevant to the resource-constrained context. Due to the dual burden of KSHV and HIV in sub-Saharan Africa, additional cases of KICS are likely, and this syndrome will contribute to the burden of early mortality in newly diagnosed HIV patients. Addressing the diagnostic and therapeutic challenges of KICS must be a part of the overall management of the HIV pandemic.

## 1. Introduction

The prevalence of Kaposi sarcoma-associated herpesvirus (KSHV), also known as human herpes virus 8 (HHV-8), is highest in sub-Saharan Africa (SSA) and coincides with a high burden of HIV [1]. KSHV is implicated in several important clinical conditions, including Kaposi sarcoma (KS), primary effusion lymphoma, multicentric Castleman disease (MCD), KSHV inflammatory cytokine syndrome (KICS), and KSHV immune reconstitution syndrome (KS-IRIS) [1,2]. The high HIV and KSHV prevalence results in a larger number of AIDS-related KSHV-associated diseases. The KSHV seroprevalence in Malawi is reported to be around 60% [3]; the estimated prevalence of HIV (adults 15–49 years old) in 2023 was 6.7% [4]. KS remains the most common malignancy among men and is second overall in Malawi, with a prevalence of 18.8 per 100,000 persons [5]. Furthermore, early mortality in HIV remains high in SSA, [6,7] and KS is an important contributor to this burden [7]. KS was found to be the attributable cause of death in over 10% of Malawian patients dying within three months of ART initiation [8]. KS is characterized by violaceous and non-blanching macules/patches, plaques, or nodules [1,9]. Extravasated blood cells are responsible for the characteristic hue of KS lesions [2]. Oral lesions are common, and KS can involve lymph nodes, the lungs, and the gastrointestinal tract. Visual diagnosis of KS alone may lead to an incorrect diagnosis [10], therefore, skin biopsy is critical to confirm the diagnosis. Pathology reveals a typical proliferation of spindle cells (KSHV-infected cells of endothelial origin) and vascular proliferation. Immunohistochemistry demonstrates KSHV latency-associated nuclear protein (LANA) in infected cells [1,9]. Paclitaxel is the first-line treatment for KS in SSA based on a regional trial showing the superiority of paclitaxel over bleomycin/vincristine and etoposide [11].

An inflammatory “MCD-like syndrome” among patients with HIV and KS (or serologic evidence of KSHV) was published in 2010 [12]. Subsequently, additional KICS cases were described in case reports and series [13,14,15,16,17,18,19,20,21,22,23,24,25,26,27,28,29,30,31,32]. Rarely, KICS has been reported in HIV-negative patients with classical KS [33]. KICS in HIV-positive patients is characterized by clinical worsening and nonspecific inflammatory symptoms, as well as elevated inflammatory serum markers, including CRP, ferritin, IL-10, IL-18, and human and viral IL-6 [12,13,24,34]. The HIV viral load is usually high and the CD4 count is typically low [17,34]. While the clinical picture and metabolomics resemble MCD, the patients lack the histological characteristics of MCD [12,13,24,34]. Lymph node biopsies often demonstrate KS partially or entirely replacing the lymph node [16]. A case definition for KICS has been proposed by Polizzotto et al. and includes clinical characteristics (fever, fatigue, edema, cachexia, respiratory symptoms, gastrointestinal disturbances, arthralgias and myalgias, altered mental status, and neuropathy), laboratory abnormalities (anemia, thrombocytopenia, hypoalbuminemia, hyponatremia), and imaging findings (lymphadenopathy, hepatosplenomegaly, effusions). Diagnosis requires the presence of ≥2 criteria from ≥2 of these categories, elevated CRP as evidence of systemic inflammation, an elevated KSHV viral load, and the absence of evidence of MCD on histopathologic review of tissue from lymph node biopsy [13,16]. Likely due to the rarity of KICS in high-income countries, high-quality clinical trial data on optimal treatments are lacking. Approaches to KICS treatment often include elements of therapy for KS and MCD [35]. Case reports proposed the early use of multidrug regimens targeting the following three processes: (1) the underlying HIV disease; (2) the KS itself; and (3) the massive inflammatory response that occurs in KICS [28].

Considering the high seroprevalence of KSHV and HIV, an elevated incidence of KICS can be assumed in SSA, likely adding to the elevated early mortality attributable to KS. Unfortunately, obtaining many of the investigations necessary for the case definition proves challenging or even impossible in many resource-constrained settings. Specifically, viral load testing for KSHV and lymph node biopsies with a full histopathological review cannot consistently be performed. Therefore, the condition is likely underrecognized, underdiagnosed, and undertreated. To add to the growing literature on KICS, highlight challenges in detection and diagnosis in resource-constrained settings, and demonstrate possible effective management within our context, we report on a case of KICS managed at the Lighthouse HIV treatment center in Lilongwe, Malawi and propose an adapted case definition of KICS for resource-constrained settings.

## 2. Case Presentation

**Figure 1:**
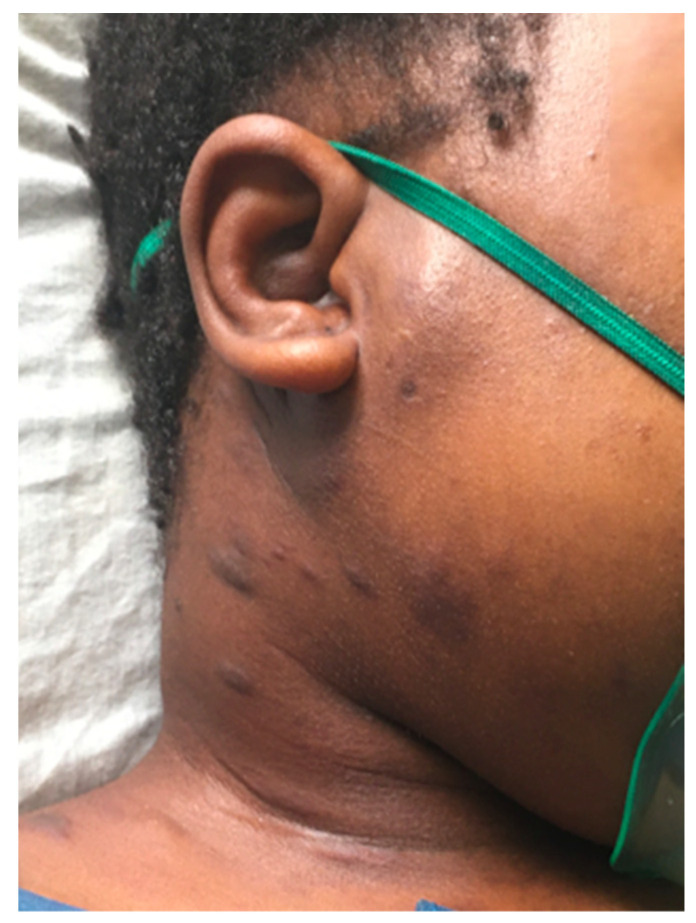
Characteristic Kaposi sarcoma lesions on the right side of the neck of the patient on presentation (Day 0).

A 19-year-old HIV-positive woman presented with severe shortness of breath and dizziness. She had been on antiretroviral therapy (ART) for 16 years and had been followed at our institution for the past two years. ART non-adherence was likely, as multiple detectable HIV viral loads (between 250–16,000 copies/mL) were recorded during these two years. Her current HIV viral load was 8000 copies/mL and CD4 count was 49 cells/µL. The patient presented in acute distress, tachycardic (heart rate 137/min), hypotensive (blood pressure 90/60 mmHg), and hypoxic with an oxygen saturation (SpO2) of 68%. The patient weighed 51 kg, and the exam was notable for facial edema and KS lesions on the neck (Figure 1).

Laboratory examinations (Table 1) revealed severe anemia with a hemoglobin of 5.4 g/dL and thrombocytopenia of 80,000/µL. Serum-cryptococcus antigen and urine-lipoarabinomannan (LAM) for disseminated tuberculosis were negative. 

**Figure 2:**
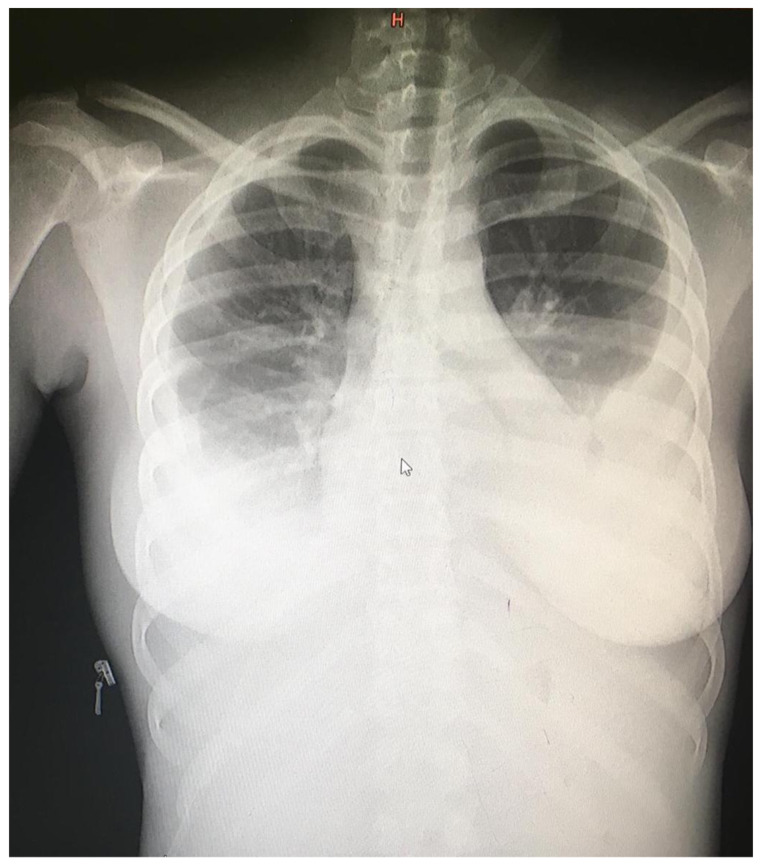
Chest radiograph (Day 0) showed bilateral pleural effusions and possible left lower-zone infiltrates.

Chest radiograph (Figure 2) showed bilateral pleural effusions and suspected left lower-zone infiltrates without evidence of pulmonary tuberculosis. She received oxygen via facemask at 10 L/min, intravenous fluids, and ceftriaxone (2 g daily for five days). Thoracentesis of the left hemithorax yielded 2 L of bloody fluid. Investigations from the pleural fluid are not routinely sent and were not performed. The patient was admitted to the adjacent referral hospital and received two units of blood, with an improvement in her hemodynamics. Details of the clinical course are summarized in Table 2.

**Figure 3:**
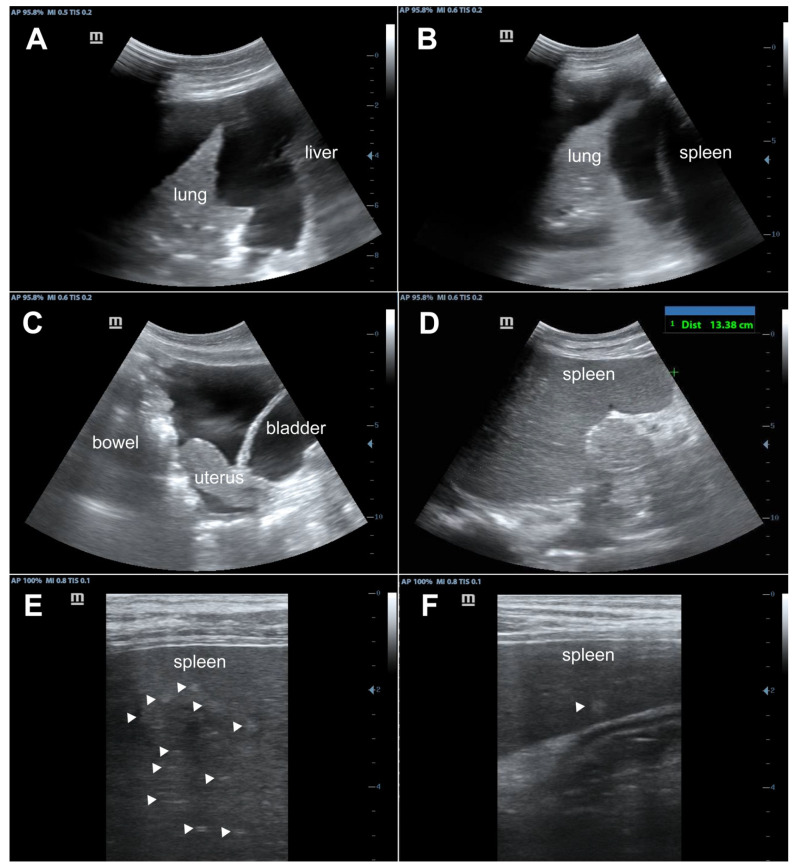
Point-of-care ultrasound performed on Day 2. Findings were consistent with disseminated KS, including right pleural effusion (**A**); left pleural effusion (**B**); pelvic free fluid (**C**); an enlarged spleen (**D**); and multiple hyperechoic splenic lesions (the arrowheads in **E**,**F**).

**Figure 4:**
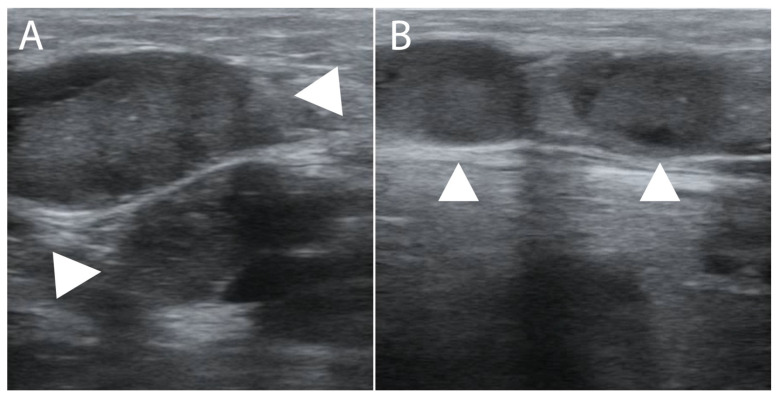
Multiple enlarged inguinal lymph nodes (arrowheads) were identified by ultrasound (**A**,**B**).

**Figure 5:**
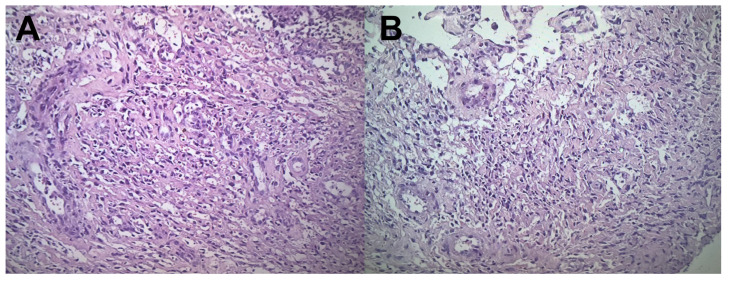
Ultrasound-guided biopsy of one of the many enlarged inguinal lymph nodes (see Figure 4) was performed on Day 3. Hematoxylin and eosin staining showed the spindle cells of Kaposi sarcoma had completely obliterated normal lymph node tissue (**A**,**B**). There was no evidence of multicentric Castleman disease.

During admission, the patient complied with the prescribed ART (tenofovir/lamivudine/dolutegravir). Point-of-care ultrasound showed bilateral pleural effusions, pelvic free fluid, an enlarged spleen, and hyperechoic splenic lesions consistent with disseminated KS (Figure 3, Video S1). The KSHV viral load at admission was found to be 50,000 copies/mL. A lymph node biopsy on a cervical lymph node prior to presentation unfortunately failed to obtain diagnostic tissue, but multiple enlarged inguinal lymph nodes were easily identified on ultrasound (Figure 4), so a repeat biopsy on an inguinal node was performed. The histopathology showed the classic spindle cell morphology of KS without evidence of MCD (Figure 5). KICS in the setting of AIDS with disseminated KS was strongly suspected; therefore, the patient was started on our standard regimen of IV paclitaxel (100 mg/m^2^). Given the hypotension, tachycardia, and general impression of possible KICS, we decided to start IV dexamethasone despite the possibility of the associated risk of a possible worsening of KS.

Over the course of the next week, supplemental oxygen was weaned, facial edema improved, her appetite returned, and the patient was discharged with plans for an oral steroid taper and paclitaxel outpatient treatment. When returning for the second cycle of paclitaxel after a two-week interval, she reported no shortness of breath and was stable enough to return to her classes. Laboratory examinations were also improved (Table 1). Given her history of questionable ART adherence, counseling was repeated. The paclitaxel treatment frequency was reduced to every three weeks. Over the course of the outpatient follow-up, she completed six paclitaxel cycles, the KS skin lesions resolved, her weight increased to 56 kg, and repeat ultrasound showed complete resolution of the pleural effusions, a normalization of spleen size to 11 cm, and resolution of the splenic lesions. Although her HIV viral load was undetectable about four months after discharge, her CD4 count remained low (Table 1). Unfortunately, we were unable to obtain a repeat KSHV viral load.

## 3. Discussion

We report a case of KICS and demonstrate that early, appropriate treatment can lead to clinical improvement and a positive outcome. Awareness of KICS in our setting remains low. To determine the training needs of providers at our clinic, we informally contacted health care workers involved in KS care in central Malawi to determine awareness and knowledge gaps around KICS. A total of 40 providers (32 clinicians and 8 oncology nurses, ages 26–59 years old) were contacted and all completed the survey (Table S1). Two-thirds (*n* = 27) had never heard of KICS and the remaining third (*n* = 13) recognized the name but reported that they did not remember enough details to be able to diagnose this clinical entity. At the same time, 85% (*n* = 34) related that they had encountered patients with a presentation suggestive of KICS, including fever, sepsis, edema, and/or hemodynamic instability. Furthermore, 63% (*n* = 25) reported postponing chemotherapy because of such a clinical presentation. Some of this lack of awareness may be due to the impossibility of fulfilling all the requirements of the existing KICS case definition, which requires a KSHV viral load, biopsy, and histopathological review.

### 3.1. Case Definition of KICS

In our patient, the clinical signs and symptoms of the case definition were fulfilled (respiratory symptoms, fatigue, and edema). Laboratory analyses showed anemia, thrombocytopenia, and hypoalbuminemia. The point-of-care ultrasound revealed lymphadenopathy, pleural effusions, and borderline splenomegaly. Therefore, our patient met > 2 criteria from >2 categories of the clinical manifestations. In addition, the CRP and KSHV viral load were elevated, and the biopsy did not show changes characteristics of MCD. Thus, our patient fully met the criteria proposed by Polizzotto and colleagues [16].

In this case, we were only able to obtain a KSHV viral load and lymph node biopsy with a pathologic review due to the temporary availability of services supported by research projects; these are rarely available in resource-constrained settings. To suggest more readily applicable diagnostic guidance for our setting and drawing on the expertise of an oncologist (MSP) and an infectious disease specialist (TH) with extensive experience working in HIV and KS care in SSA, we propose an adaptation to the previously published KICS case definition (Table 3). Our recommended presumptive adult case definition includes a confirmation of KS but does not rely on the KSHV viral load or lymph node pathology, as these are frequently unavailable. Dependence on these two investigations to diagnose KICS likely results in under-detection in resource-constrained settings. We made these two criteria optional in our definition of presumptive KICS. Confirmation of KS remains important and can be accomplished by skin biopsy. Other authors have previously noted the difficulty in obtaining the investigations necessary to meet the Polizzotto et al. definition [19,35,36]. For example, KICS has been described in a cohort of children in Lilongwe, and diagnostic criteria relevant to both pediatrics and the resource-constrained setting were developed. These pediatric criteria include the following: lymphadenopathy > 6 cm in diameter; thrombocytopenia < 50,000 cells; hemoglobin < 8 g/dL; persistent temperature > 38 °C; and massive hepatosplenomegaly on exam [19].

### 3.2. Differential Diagnosis of KICS

KICS represents a state of immune dysregulation that generally overlaps with a variety of other syndromes, which may often be difficult to distinguish from each other. KICS can overlap with other disseminated processes among HIV patients in our setting such as disseminated MTB, lymphoma or other malignancies, disseminated fungal infections, bacillary angiomatosis, as well as renal, cardiac, or hepatic failure [36,37,38]. The presentation of KICS may also overlap with hemophagocytic lymphohistiocytosis [26]. In our definition of presumed KICS, we emphasize the importance of ruling out other mimics of KICS, most notably disseminated MTB and sepsis, as completely as possible given the available diagnostic tests. We recommend the focused assessment with sonography for HIV-associated tuberculosis (FASH) point-of-care ultrasound protocol as the initial imaging modality to exclude typical findings of disseminated TB, reveal findings consistent with disseminated KS, and thereby to support the diagnosis of KICS [37,39]. The FASH assesses for findings that increase the probability of disseminated MTD, including pericardial effusions, unilateral pleural effusions, ascites, enlarged abdominal lymph nodes, and hypoechoic splenic micro-abscesses. While several of the findings overlap with KS, the presence of hypoechoic micro-abscesses can help to differentiate disseminated TB from KS; therefore, these must be absent in our definition (Table 3).

Other KSHV-associated diseases must also be considered. KSHV-associated MCD is a polyclonal lymphoproliferative disorder characterized by systemic inflammation, lymphadenopathy, and characteristic pathologic findings [2,9]. The symptoms overlap with KICS, and patients typically have a high KSHV viral load and elevated inflammatory markers, including IL-10 and vIL-6. KICS has even been described as a “liquid” variant of MCD [31]. Nevertheless, MCD patients often show relatively well-controlled HIV with a suppressed HIV viral load and CD4 cells > 200 cells/µL [17,34]. Rituximab, thought to deplete KSHV-infected B-cells, is the first-line treatment for KSHV-associated MCD [35]. KS-IRIS refers to worsening KS and overall clinical deterioration after initiation of ART [22,40,41]. While the defining criteria of KS-IRIS are variable, key elements include a worsening of underlying KS within the first twelve weeks following ART initiation in the setting of evidence of viral suppression and an increase in CD4 count [42]. The following four factors have been associated with risk of developing KS-IRIS: detectable KSHV viral load; a hematocrit < 30%; a higher HIV viral load; and prior treatment of KS [43]. The temporal correlation with the initiation of ART is usually helpful in the diagnosis. In our case, both KS-IRIS and MCD were considered. Although the patient had not been perfectly adherent to her ART prior to her hospitalization and was continued on ART without delay, two factors make KS-IRIS less likely. First, she presented critically ill before the effect of more closely monitored ART could have been realized, and second, the CD4 count remained low throughout her course. Although the patient did have lymphadenopathy, the biopsy did not reveal histopathological findings typical for MCD. Therefore, we concluded KICS to be the most likely diagnosis.

### 3.3. Management of KICS in Resource-Constrained Settings

KICS is a serious disease with potentially high mortality; therefore, treatment should be started promptly and address the underlying HIV disease, KS, and the inflammatory response [28]. The treatment of HIV is necessary to induce control of the KSHV infection, but it may be inappropriately delayed for fear of precipitating KS-IRIS. Direct treatment of KS is necessary because patients usually present with disseminated KS disease that ART alone will be unlikely to control. In addition, KS spindle cells are at least partially responsible for the inflammatory milieu of KICS (e.g., via production of vIL-6). Again, paclitaxel is the first-line treatment for KS in our setting [44]. In high-income settings, liposomal doxorubicin may be used in lieu of paclitaxel; additional therapies currently under investigation include immune checkpoint inhibitors, proteosome inhibitors, and cytokine-based treatments such as IL-7 and IL-12 [35]. Given the role of inflammasome activation, additional targets may include IL-18 and IL-1β [34]. Direct treatment of KS may be inappropriately delayed for fear that the patient is too sick to tolerate chemotherapy; however, other cases also provide examples showing that early treatment can be associated with positive outcomes [28]. Furthermore, directly treating the hyperimmune state is necessary because the immune response is responsible for the elements of critical illness, such as distributive shock and acute respiratory distress syndrome, which carry a high risk of acute mortality. However, treatments (e.g., rituximab, steroids) may be withheld for fear of worsening the KS disease and increased short-term mortality [45,46]. In our case, we were able to achieve the three pillars of treatment for KICS with ART, paclitaxel, and steroids [44]. We recognize the abovementioned dangers of these treatments but felt the potential benefit to outweigh these risks in our patient.

### 3.4. Suggestions for Future Research

Several questions require further investigation. Especially in SSA, the prevalence of KICS needs to be better delineated using case definitions that are sensitive to the local context. When biopsies and pathology are available, standardized criteria for ruling out MCD should be further elucidated; defining for example, the number of biopsy attempts considered sufficient to rule out MCD would be helpful. In addition, if adequate immunohistochemistry is not available for LANA, a pathologic diagnosis of MCD is quite difficult. The optimal agents, doses, and duration of treatment need to be defined, especially in the case of steroids. Furthermore, clinical trials assessing the timing and duration of different treatment modalities are needed to optimize therapeutic regimens while minimizing the risks of KS-IRIS or worsening KS disease.

## 4. Conclusions

Given the dual burden of KSHV and HIV in Malawi, we suspect there are far more cases of KICS than may be identified and reported. Clinicians may have encountered KICS before, but most are unaware of the entity and how to make the diagnosis. While a case definition has been proposed, not all the required investigations are routinely available in resource-constrained contexts. The three pillars of treatment for KICS include the following: treatment of the underlying HIV; direct treatment of KS; and addressing the hyper-inflammatory response. By proposing a case definition for presumed KICS applicable to our setting and demonstrating a potential efficacious treatment regimen using commonly available medications, we hope that the identification and treatment of KICS can be improved.

## Figures and Tables

**Table 1:** Results of laboratory examinations during the patient’s hospital course and outpatient follow-up. KSVH: Kaposi sarcoma-associated herpesvirus, UD: undetectable.

	Reference Range	Day 0	Day 2	Day 4	Day 17	Day 39	Day 59	Day 116
**White-cell count** (per µL)	4000–10,000	5500	3400		2700	3400	3600	2000
**Hemoglobin** (g/dL)	10.9–17.3	5.4	5.2	6.9	9.8	11.1	11.9	15.9
**Mean corpuscular volume** (fl)	71–95	92	94		97	89	93	94
**Platelet count** (per µL)	122–330	80,000	35,000		100,000	185,000	158,000	158,000
**Urea nitrogen** (mg/dL)	18–55		100		19		17	
**Creatinine** (mg/dL)	0.7–1.3		1.80	0.80	0.50	0.53	1.10	1.00
**Alanine aminotransferase** (U/L)	0–45		33		13			
**Aspartate aminotransferase** (U/L)	0–35		12		11		13	16
**Alkaline phosphatase** (U/L)	64–304		48					
**Gamma-glutamyl transferase** (U/L)	0–55		20					
**Total bilirubin** (mg/dL)	6.8–8.3		0.46		1.50		0.20	0.75
**Total protein** (g/dL)	6.8–8.3		5.5					8.1
**Albumin** (g/dL)	3.5–5.2		2.1					4.8
**C-reactive protein** (mg/L)	<1		20.6			6.4		3.4
**CD4** (per µL)	500–1500	49				53		69
**HIV viral load** (copies per mL)	UD	8000				975		UD
**KSVH viral load** (copies per mL)	UD		50,000					

**Table 2:** Case timeline.

**Day 0**	**Patient presented hypotensive and hypoxic. Left thoracentesis removed 2000 mL bloody fluid. Requiring supplemental oxygen started at 10 L/min via facemask. Received intravenous fluids, ceftriaxone 2 g, two units of blood.**
**Day 1**	Hemodynamics and respiratory status improved. Oxygen saturation 97% on 5 L/min. Received second dose ceftriaxone. KICS suspected.
**Day 2**	Ultrasound revealed bilateral pleural effusions, pelvic free fluid, and hyperechoic splenic lesions consistent with disseminated KS. Right thoracentesis removed 1300 mL blood-stained fluid. Received first cycle of IV paclitaxel 100 mg/m^2^, IV dexamethasone 12 mg, third dose of ceftriaxone 2 g.
**Day 3**	Received dexamethasone 12 mg, two units of blood, and fourth dose ceftriaxone 2 g. Inguinal lymph node biopsy performed.
**Day 4**	Received dexamethasone 8 mg and last dose of ceftriaxone 2 g.
**Day 5**	Prednisone 40 mg.
**Day 6**	Prednisone 40 mg.
**Day 7**	Prednisone 40 mg. Discharged from hospital.
**Day 8–14**	Prednisone 30 mg daily at home.
**Day 15–21**	Prednisone 15 mg at home.
**Day 17**	Clinic visit for paclitaxel cycle 2 with repeat laboratory examinations. Received ART adherence counseling. Patient felt improved and wanted to return to school.
**Day 39**	Clinic visit for paclitaxel cycle 3 with repeat laboratory examinations and ultrasound. Reported adherence to ART regimen.
**Day 59**	Clinic visit for paclitaxel cycle 4 with repeat laboratory examinations.
**Day 80**	Clinic visit for paclitaxel cycle 5.
**Day 101**	Clinic visit for paclitaxel cycle 6.
**Day 116**	Post treatment follow up visit in clinic with repeat laboratory examinations and ultrasound. Patient felt well and was back in school.

**Table 3:** Presumptive KICS case definition for resource-constrained settings. Adapted from Polizzotto et al. with differences in bold [16]. Our context-adapted presumptive KICS case definition requires the presence of at least 2 clinical manifestations drawn from at least 2 categories (1a, b, and c), together with each of the criteria in 2 and 3. Optional findings support the diagnosis but can rarely be performed in resource-constrained settings. Criteria met by our patient are marked with an asterisk. KS: Kaposi sarcoma, MTB: *Mycobaterium* tuberculosis, LAM: lipoarabinomannan, FASH: focused assessment with sonography for HIV-associated tuberculosis, KSHV: Kaposi sarcoma-associated herpesvirus, MCD: multicentric Castleman disease.

**1. Clinical manifestations**	a. Symptoms and signs – Fever – * Fatigue – * Edema – * Cachexia – * Respiratory symptoms – Gastrointestinal disturbance – Arthralgia and myalgia – Altered mental state – Neuropathy with or without pain b. Laboratory abnormalities – * Anemia – * Thrombocytopenia – * Hypoalbuminemia **(if available)** – Hyponatremia **(if available)**	c. Radiographic abnormalities – * Lymphadenopathy – * Splenomegaly – Hepatomegaly – * Body cavity effusions – * Focal KS lesions in the liver and/or spleen
**2. No strong evidence for other source of systemic inflammation**	**a** * KS lesions without evidence of bacterial superinfection **b** * No other bacterial, fungal, or parasitic infection evident **c** * No evidence of disseminated MTB: negative urine-LAM test, negative GeneXpert^®^ MTB, FASH ultrasound without typical hypoechoic spleen lesions **d** * Treatment with broad-spectrum antibiotic for ≥ 5 days without success (Note, presumptive diagnosis can be made before antibiotic course has been completed)	
**3. Confirmation of KS**	**a** * Characteristic findings of KS on skin biopsy **(or lymph node core needle biopsy, if available)**	
**Optional findings** **(usually not available)**	* Elevated C-reactive protein ≥ 3 g/dL * Elevated KSHV viral load in plasma ≥ 1000 copies/mL or peripheral blood mononuclear cells ≥ 100 copies/106 cells as evidence of KSHV activity * Absence of KSHV-associated MCD on histopathologic assessment of lymphadenopathy	

## Data Availability

All available data relevant to this case have been included in the manuscript.

## Supplementary Materials

The following supporting information can be downloaded at: https://www.mdpi.com/article/10.3390/tropicalmed9120307/s1, Table S1: Health worker surveys; Video S1: Hyperechoic splenic lesions.
