# Supplementary material for: Diagnosis and Management of Kaposi Sarcoma-Associated Herpesvirus Inflammatory Cytokine Syndrome in Resource-Constrained Settings: A Case Report and an Adapted Case Definition
Source: Trop Med Infect Dis. 2024 Dec 16;9(12):307. doi: 10.3390/tropicalmed9120307 (PMC11680326; doi:10.3390/tropicalmed9120307)
Supplement: Supplementary file 1 [file tropicalmed-09-00307-s001.zip › tropicalmed-3308869-supplementary.pdf]

Table S1: Provider Survey

Age: _____ Gender: ☐F ☐M

Hospital or clinic: ______________________________________

1. What does best describe your cadre?
   ☐ Clinical officer ☐ Medical officer ☐ Specialist ☐ Nurse

2. Which of the three describe your current main field of work best?
   ☐ HIV/ART medicine ☐ Oncology ☐ General medicine (with HIV and Onco)

3. How many Kaposi sarcoma (KS) patients have you cared for **or** diagnosed **or** treated in the last 12 months?
   ☐ 0 ☐ 1-5 ☐ 6-10 ☐ 10-20 ☐ more than 20

4. Have you ever seen patients with KS that were severely sick, seemingly septic, with fever, widespread edema and maybe even shock (low blood pressure and high heart rate). Possibly they also had respiratory problems or altered mental status or large effusions?
   ☐ never or cannot remember
   ☐ yes, but very rare
   ☐ yes, we do see them from time to time

5. Have you (and your team) ever decided not to give chemo (BV or paclitaxel) because the patient seemed "too sick" (like described above)?
   ☐ no ☐ yes ☐ does not apply/do not remember

6. Have you ever heard of KICS (Kaposi Sarcoma Virus Inflammatory Cytokine Syndrome)?
   ☐ no
   ☐ yes

6a. If you have heard of KICS, how would you best describe your knowledge?
   ☐ I heard of it but do not remember any details
   ☐ I heard of it and remember some signs and symptoms, but I could not diagnose it
   ☐ I know it and I would know how to diagnose and maybe even treat it
